# Retinal Neuroprotection From Optic Nerve Trauma by Deletion of Arginase 2

**DOI:** 10.3389/fnins.2018.00970

**Published:** 2018-12-20

**Authors:** Zhimin Xu, Abdelrahman Y. Fouda, Tahira Lemtalsi, Esraa Shosha, Modesto Rojas, Fang Liu, Chintan Patel, R. William Caldwell, Subhadra Priya Narayanan, Ruth B. Caldwell

**Affiliations:** ^1^Charlie Norwood VA Medical Center, Augusta, GA, United States; ^2^Vascular Biology Center, Augusta University, Augusta, GA, United States; ^3^James and Jean Culver Vision Discovery Institute, Augusta University, Augusta, GA, United States; ^4^Department of Pharmacology and Toxicology, Augusta University, Augusta, GA, United States; ^5^Program in Clinical and Experimental Therapeutics, University of Georgia, College of Pharmacy, Augusta, GA, United States; ^6^Department of Cell Biology and Anatomy, Augusta University, Augusta, GA, United States; ^7^Department of Ophthalmology, Augusta University, Augusta, GA, United States

**Keywords:** arginase 2, retina, neuroprotection, optic nerve crush, brain derived neurotrophic factor, retinal ganglion cells

## Abstract

Our previous studies have implicated expression of the mitochondrial isoform of the arginase enzyme arginase 2 (A2) in neurovascular injury during ischemic retinopathies. The aim of this study was to characterize the specific involvement of A2 in retinal injury following optic nerve crush (ONC). To accomplish this, wild-type (WT) or A2 knockout (A2^-/-^) mice were subjected to ONC injury. The contralateral eye served as sham control. Quantitative RT-PCR and western blot were used to evaluate mRNA and protein expression. Retinal ganglion cell (RGC) survival was assessed in retinal whole mounts. Axonal sprouting was determined by anterograde transport of Cholera Toxin B (CTB). These analyses showed increased A2 expression following ONC. Numbers of NeuN-positive neurons as well as Brn3a- and RBPMS-positive RGC were decreased in the WT retinas at 14 days after ONC as compared to the sham controls. This ONC-induced neuronal loss was diminished in the A2^-/-^ retinas. Similarly, axonal degeneration was ameliorated by A2 deletion whereas axon sprouting was enhanced. Significant retinal thinning was also seen in WT retinas at 21 days after ONC, and this was blocked in A2^-/-^ mice. Cell death studies showed an increase in TUNEL positive cells in the RGC layer at 5 days after ONC in the WT retinas, and this was attenuated by A2 deletion. ONC increased glial cell activation in WT retinas, and this was significantly reduced by A2 deletion. Western blotting showed a marked increase in the neurotrophin, brain derived neurotrophic factor (BDNF) and its downstream signaling in A2^-/-^ retinas vs. WT after ONC. This was associated with increases in the axonal regeneration marker GAP-43 in A2^-/-^ retinas. Furthermore, A2^-/-^ retinas showed decreased NLRP3 inflammasome activation and lower interleukin (IL-) 1β/IL-18 levels as compared to WT retinas subjected to ONC. Collectively, our results show that deletion of A2 limits ONC-induced neurodegeneration and glial activation, and enhances axonal sprouting by a mechanism involving increases in BDNF and decreases in retinal inflammation. These data demonstrate that A2 plays an important role in ONC-induced retinal damage. Blockade of A2 activity may offer a therapeutic strategy for preventing vision loss induced by traumatic retinal injury.

## Introduction

Impaired vision secondary to optic nerve damage is a common and often unrecognized complication of numerous ophthalmologic and neurologic conditions. Traumatic ocular injury is frequently associated with degeneration of retinal ganglion cells (RGC) due to primary trauma to their axons that travel through the optic nerve to the brain. This may also involve degeneration of other retinal neurons secondary to oxidative stress, vascular dysfunction, ischemia and edema, which can eventually cause permanent vision loss (Weber et al., 2008). So far, there is no effective treatment for optic nerve trauma, because of the lack of understanding of the detailed molecular mechanisms by which the retinal neurons are damaged. The mouse model of optic nerve crush (ONC) injury has been widely used to study axonal degeneration and the subsequent RGC and neuronal losses characteristic of traumatic optic neuropathy (TON) and glaucoma induced optic nerve degeneration (Tang et al., 2011). Several pathways have been studied as molecular targets to limit neurodegeneration and enhance repair after ONC. One of the most extensively studied molecules in this context is the neurotrophin brain derived neurotrophic factor (BDNF). BDNF has been shown to protect RGC and their axons against injury via its downstream survival signaling pathways (Mysona et al., 2017) Therefore, there is a great interest in developing therapies that enhance BDNF either directly by increasing its mRNA/protein levels (e.g., via gene therapy) or indirectly by activating pathways that lead to BDNF upregulation (Ratican et al., 2018).

Our group has been investigating the role of the arginase pathway in neurovascular injury during retinopathy. Arginase is a ureohydrolase enzyme that converts L-arginine to urea and ornithine. There are two isoforms of the arginase enzyme, arginase 1 (A1) and arginase 2 (A2). Both isoforms are known to be involved in the pathophysiology of different central nervous system disorders including retinal disease (Caldwell et al., 2018). We have shown that the mitochondrial isoform, A2, is critically involved in the neurovascular injury associated with retinal ischemia-reperfusion injury (Shosha et al., 2016). In this model, A2 deletion protected against RGC and microvascular degeneration via decreased glial activation, oxidative stress and cell death by necroptosis (Shosha et al., 2016). On the other hand, we have recently shown that A1 is neurovascular protective in the same model via ameliorating macrophage inflammatory response (Fouda et al., 2018). In the current study, we examined the role of A2 in TON-induced retinal neuronal injury using A2^-/-^ mice subjected to ONC. We also examined the possible cross talk between A2 and BDNF which has been extensively studied in the ONC model. We also assessed potential involvement of BDNF down-stream targets including extracellular regulated and mitogen- and stress-activated protein kinases (ERK/MSK) and growth associated protein (GAP)-43 (Gupta et al., 2009), which is induced after ONC injury (Doster et al., 1991) and is thought to be involved in axonal regeneration (Holahan, 2017).

## Materials and Methods

### Animals and Optic Nerve Crush Induction

**FIGURE 1:**
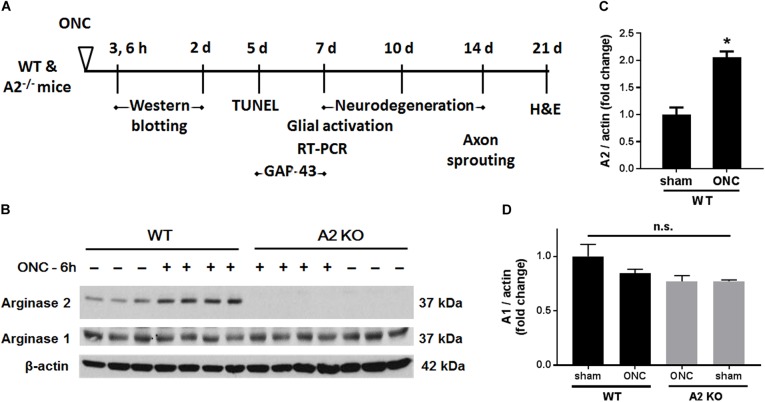
A2 is increased in retinas subjected to ONC injury. Schematic diagram **(A)** of the different time points examined after subjecting WT and A2^-/-^ mice to ONC. Western blotting **(B)** of retinal lysates for A1 and A2 protein expression and quantification **(C,D)** showed increases in A2 in response to ONC at 6 h after injury. A2^-/-^ retinas lacked the A2 protein band. A1 expression was similar in all groups. *N* = 3–4, ^∗^*p* < 0.01 vs. WT sham, n.s. means no statistically significant difference.

Male Wild-type C57BL/6J (WT) and Arginase-2 knockout (A2^-/-^) mice on C57BL/6J background were maintained in our animal facility and used to study ONC injury. All animal procedures performed in this study complied with the ARVO statement for the use of Animals in Ophthalmic and Vision Research. All surgeries were performed under anesthesia and analgesia was provided to minimize suffering. For induction of ONC, mice (10–12 weeks old) were anesthetized by injection of Ketamine and Xylazine cocktail intraperitoneal and one drop of topical anesthesia (0.5% proparacaine hydrochloride) was applied to the eyeball and the surrounding intraocular muscles before the surgery. Briefly, the conjunctiva on the left eye was incised, the orbital muscles were deflected and the optic nerve 1–2 mm from the eyeball was clamped for 3 s using self-closing N7 forceps (Fine Science Tools, Foster City, CA, United States). The right eye was used as sham control. Mice were injected subcutaneously with buprenorphine for analgesia, returned to their home cage, and monitored until recovery (Tang et al., 2011). Eyes and retinas were harvested for analysis at various times from 3 h to 28 days after ONC. The design for these analyses is shown in Figure 1A.

### Neurodegeneration and Ganglion Cell Survival Evaluation

NeuN (neuronal nuclei) was used as a neuronal cell marker to label surviving neurons in retinal flat mounts. Brn3a (brain-specific homeobox/POU domain protein 3A) and RBPMS (RNA binding protein, mRNA processing factor) were used as RGC markers (Nadal-Nicolas et al., 2009; Rodriguez et al., 2014). Eyeballs were collected 7, 10, or 14 days after ONC and fixed in 4% paraformaldehyde (PFA) at 4°C overnight. Isolated retinas from these eyeballs were incubated with anti-NeuN, anti-Brn3a, or anti-RBPMS (Table 1) in 37°C for 2 h and then incubated overnight with fluorescein-conjugated secondary antibodies (Invitrogen, Carlsbad, CA; 1:400). Retinal flat mounts were imaged using a confocal microscope (LSM 780; Carl Zeiss, Thornwood, NY, United States). Four images were taken in the mid-periphery of the ganglion cell layer (GCL) of each retina and the NeuN, Brn3a, or RBPMS positive cells were counted using ImageJ. Data are presented as percent of NeuN, Brn3a, or RBPMS positive cell numbers in the GCL of WT sham retinas.

**Table 1:** Antibodies.

Antibody	Catalog number	Company	Dilution	Experiment
NeuN	MAB377	Millipore, Billerica, MA	1:200	Immunostaining
Brn3a	SC-31984	Santa Cruz, Dallas, TX	1:200	Immunostaining
RBPMS	ABN1362	Millipore, Billerica, MA	1:200	Immunostaining
TUJ1	801202	BioLegend, San Diego, CA	1:200	Immunostaining
GAP-43	GTX30199	GeneTex, Irvine, CA	1:200	Immunostaining
GFAP	Z0334	Dako, Carpinteria, CA	1:200	Immunostaining
Iba1	019-19741	Wako, Richmond, VA	1:200	Immunostaining
ICAM-1	SC-1511	Santa Cruz, Dallas, TX	1:500	Western blotting
Arginase 1	SC-20150	Santa Cruz, Dallas, TX	1:500	Western blotting
Arginase 2	SC-20151	Santa Cruz, Dallas, TX	1:500	Western blotting
BDNF	SC-546	Santa Cruz, Dallas, TX	1:1000	Western blotting
p-ERK1/2	4370	Cell Signaling, Danvers, MA	1:500	Western blotting
total ERK1/2	4695	Cell Signaling, Danvers, MA	1:500	Western blotting
p-MSK1	9595	Cell Signaling, Danvers, MA	1:500	Western blotting
total MSK1	3489	Cell Signaling, Danvers, MA	1:500	Western blotting
NLRP3	15101	Cell Signaling, Danvers, MA	1:500	Western blotting
IL-1β	AF-401-NA	R&D Systems, Minneapolis, MN	1:1000	Western blotting
β-actin	4511	Sigma-Aldrich, St. Louis, MO	1:5000	Western blotting


### Nerve Fiber Degeneration and Sprouting

TUJ1 (Neuron-specific class III beta-tubulin) was used as a nerve fiber marker and GAP-43 (growth associated protein 43) was used as an axonal regeneration marker in retinal flat mounts. Eyeballs were collected 7 days (for TUJ1 and GAP-43) or 14 days (for TUJ1) after ONC and fixed in 4% PFA at 4°C overnight. Isolated retinas from these eyeballs were labeled with anti-TUJ1 or anti-GAP-43 (Table 1) at 37°C for 2 h and then incubated overnight with Alexa Fluor 488 conjugated goat anti–mouse IgG2a or Alexa Fluor 594 conjugated goat anti–rabbit antibodies (Invitrogen, Carlsbad, CA, United States; 1:400) at 4°C for overnight. Retinal flat mounts were imaged using fluorescence microscopy (Axioplan2; Carl Zeiss, Thornwood, NY, United States) or confocal microscope (LSM 780; Carl Zeiss, Thornwood, NY, United States). For quantifying total numbers of surviving nerve fibers, retinal whole-mount images were taken and the TUJ1 positive fibers were counted at a distance 1/3 of the retina radius starting from optic nerve head using ImageJ. The results are presented as a percent of TUJ1 positive nerve fibers in the GCL of the ONC eyes compared to the sham eyes.

For analysis of axonal sprouting, mice were subjected to 1 s-ONC and sprouting axons were identified by anterograde labeling with Alexa Fluor^®^ 647-conjugated Cholera Toxin B (CTB; ThermoFisher, cat. # C34778, Waltham, MA, United States) as described previously (Wang et al., 2018). Duration of 1 s crush was selected for this experiment vs. 3 s for others based on preliminary studies that showed more sprouting after 1 s crush. Briefly, the mice were anesthetized at 12 days after ONC and injected intravitreally with CTB (0.2%, 2 μg/eye). After 2 days, the mice were anesthetized and perfused transcardially with phosphate buffered saline (PBS) followed by 4% PFA in phosphate buffer (pH 7.3). Optic nerves were dissected, post-fixed in 4% PFA in PBS overnight, and cleared with FocusClear^TM^ (CelExplorer, cat. # FC-101, Hsinchu, Taiwan) until transparent. Chambers were constructed on glass slides to allow space for the entire nerve thickness and prevent flattening of the nerve. The nerves were mounted using MountClear^TM^ (CelExplorer, cat. # MC-301, Hsinchu, Taiwan), the slides were coverslipped, and sprouting axons were imaged using confocal microscopy (LSM 780; Carl Zeiss, Thornwood, NY, United States). Optical slices scanned by confocal microscope were used for counting the numbers of axons at 100 μm intervals beginning at 200 μm and extending to 1 mm away from the crush site of the optic nerves. Images were presented with pseudocolor green for better observation.

### Retinal Thickness Measurement

Retinal thickness was measured on retinal sections collected 21 days after ONC injury as previously described (Shosha et al., 2016). Cross sections (10 μm thick) stained using hematoxylin and eosin (H&E) were analyzed to assess alterations in retinal morphology. Retinal images were taken at 162 μm away from the optic nerve head and two sections 20 μm apart were used for each sample. Thicknesses of whole retina at three different points per image were measured using ImageJ. Average values of thickness are presented as percentage of the values for the sham control eyes.

### Detection of Glial Activation

Seven days after ONC injury, eyeballs were collected and snap frozen in optimal cutting temperature (OCT) compound or fixed in 4% PFA for later use. The snap-frozen eyeballs were processed to prepare cryostat sections (10 μm) and labeled with anti-glial fibrillary acidic protein (GFAP) antibody to detect glial activation. The PFA fixed eyeballs were prepared for either retinal flat mounts or frozen sections as described previously (Shosha et al., 2016). Retinal flat mounts or sections were incubated with anti-ionized calcium-binding adapter molecule 1 (Iba1) antibody to label microglia/macrophages. Sections or flat mounts were then incubated with fluorescein-conjugated secondary antibodies (Invitrogen, Carlsbad, CA; 1:400) as previously described (Shosha et al., 2016). After washing with PBS, sections or flat mounts were preserved with mounting medium (Vector Laboratories Cat. # H-1000, Burlingame, CA, United States) and images were taken using a fluorescent or confocal microscope. For quantification, fluorescence intensities of GFAP or Iba1 flat mount staining images were analyzed using ImageJ software.

### TUNEL Assay

Apoptotic cells were studied in retinal samples collected 5 days after ONC using TUNEL (Terminal deoxynucleotidyl transferase dUTP nick end labeling) assay on cryosections from snap-frozen eyeballs. Fluorescein *In Situ* Cell Death Detection kit (Millipore cat. #S7110, Billerica, MA, United States) was used according to the manufacturer’s protocol. Two sections per animal were used to collect the images and the quantification of TUNEL positive cells was performed manually on the whole retinal section.

### Quantitative RT-PCR

Total RNA was extracted from retinal samples and reverse-transcribed as previously described (Shosha et al., 2016). ABI StepOne Plus Thermocycler (Applied Biosystems, Foster City, CA, United States) was used to perform quantitative PCR with master mix (Power SYBR Green, Invitrogen). Primer sequences for mouse transcripts are shown in Table 2. Data were normalized to hypoxanthine phosphoribosyltransferase (HPRT) and the fold change between levels of different transcripts was calculated by the ΔΔC*_T_* method.

**Table 2:** RT-PCR primers.

Gene name	Forward primer	Reverse primer
ICAM1	CAGTCCGCTGTGCTTTGAGA	CGGAAACGAATACACGGTGAT
IL1β	TGCCACCTTTTGACAGTGATG	ATGTGCTGCTGCGAGATTTG
IL18	TCAAAGTGCCAGTGAACCCC	GGTCACAGCCAGTCCTCTTAC
HPRT	GAAAGACTTGCTCGAGATGTCATG	CACACAGAGGGCCACAATGT


### Western Blotting

Retinal proteins were extracted using RIPA buffer (Millipore) containing protease and phosphatase inhibitors (Complete Mini and phosSTOP Roche Applied Science, Indianapolis, IN, United States). Protein lysates were then separated on SDS-PAGE and transferred to nitrocellulose membranes (Millipore), blocked in 5% milk (Bio-Rad, Hercules, CA, United States) or 2% BSA (Gemini Bio-products) in Tris-buffered saline with 0.05% Tween-20 (TBS-T). The membranes were incubated overnight (4°C) with primary antibodies against: arginase 1 and 2, p-MSK1, total MSK1, p-ERK1/2, total ERK1/2, BDNF, ICAM-1, NLRP3, IL-1β, and β-actin as listed in Table 1. The following day, horseradish peroxidase-conjugated secondary antibodies (GE Healthcare Bio-Science Corp., Piscataway, NJ, United States; 1:2000) were applied to the membranes and the protein expression levels were detected using the enhanced chemiluminescence system (GE Healthcare Bio-Science Corp., Piscataway, NJ, United States). Data were quantified by densitometry using ImageJ and normalized to β-actin as loading control.

### Statistical Analysis

GraphPad Prism 7 (GraphPad Softwar Inc., La Jolla, CA, United States) was used for statistical analysis. Two-way ANOVA followed by Tukey test was used for multiple comparisons. The student’s *t*-test (Two-tailed) was used for single comparisons. A value of *p* < 0.05 was considered statistically significant. Results are presented as mean ± SEM.

## Results

### A2 Deletion Improves Neuronal Survival in the Retinal Ganglion Cell Layer After ONC

**FIGURE 2:**
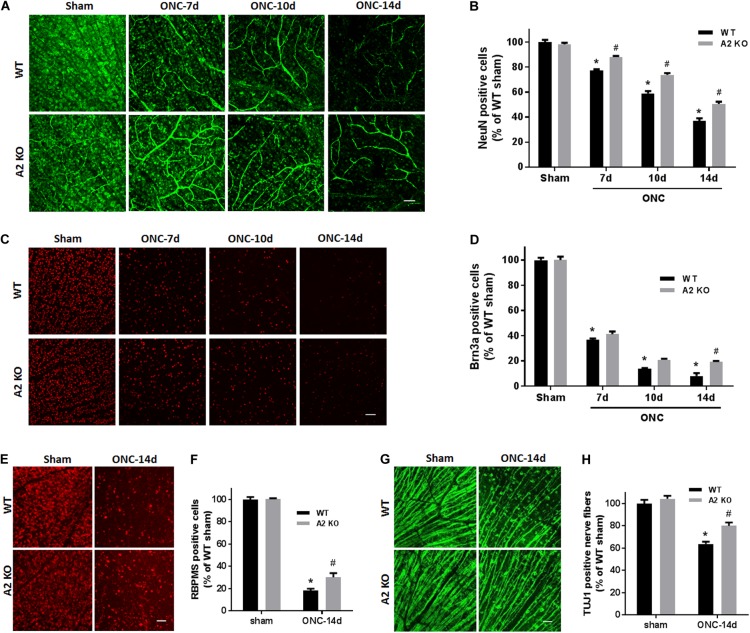
A2 deletion ameliorates ONC-induced neuronal cell loss. Representative NeuN labeling **(A)** and quantification **(B)** of retinal neurons in the GCL of retinal flat mounts show a progressive decline in NeuN^+^ cells over 14 days after ONC. A2 deletion ameliorates the ONC-induced neuronal cell loss. Immunolabeling **(C)** and quantification **(D)** for the RGC marker, Brna3a, showed a similar trend with reduced neurodegeneration in the A2^-/-^ retinas. *N* = 4–6 per group, ^∗^*p* < 0.01 vs. WT sham, ^#^*p* < 0.01 vs. A2^-/-^ sham and respective WT ONC time point. Flat mount labeling for another RGC marker, RBPMS, at 14 days **(E)** and quantification **(F)** showed similar results with significant neuroprotection in A2^-/-^ retinas. Flat-mount labeling of the retinal neurons nerve fibers using TUJ1 **(G)** and quantification **(H)** showed nerve fiber loss at 14 days after ONC. A2 deletion was associated with relative preservation of nerve fibers as compared to WT after ONC. *N* = 5–6, ^∗^*p* < 0.01 vs. WT sham, ^#^*p* < 0.01 vs. A2^-/-^ sham and WT ONC, scale bar = 50 μm.

To examine the change in A2 expression in response to ONC injury, we subjected WT and A2^-/-^ mice to ONC and collected the retinas after 6 h. Western blotting on retina lysates showed upregulation of A2 expression as compared to shams, thus suggesting the involvement of A2 in ONC pathology (Figures 1B,C). At the same time point, A1 expression was not changed by the injury or A2 deletion (Figure 1D). ONC injury has been shown to induce progressive neuronal cell loss starting at day 7 (Liu et al., 2014). To assess the potential involvement of A2 in this neuronal cell loss, we collected retinas from WT and A2^-/-^ mice at 7, 10, and 14 days after injury. Confocal imaging of the ganglion cell layer (GCL) in retina flat mounts labeled for the general neuronal marker NeuN, showed a progressive decrease in NeuN-positive neurons over time in both WT and A2^-/-^ retinas. However, A2^-/-^ retinas exhibited better preservation of neuronal cell density compared to WT retinas at the three time points examined (Figures 2A,B). Furthermore, we labeled the retina flat mounts for Brn3a, a marker for RGC. Similar to NeuN, Brn3a staining showed a progressive decline in Brn3a-positive cells over time. A2^-/-^ retinas showed significantly higher numbers of Brn3a-positive cells at 14 days compared to WT retinas (Figures 2C,D) and this was further confirmed using another RGC marker, RBPMS (Figures 2E,F). Since RGC loss after ONC happens due to damage and degeneration of the nerve fibers, we also quantified the nerve fibers in the nerve fiber layer (NFL) in flat mounts labeled with the RGC cell body and axon marker TUJ1 as previously described (Mesentier-Louro et al., 2017). WT ONC retina flat mounts showed reduced RGC nerve fibers at 14 days. A2 deletion significantly protected against the ONC-induced loss of the RGC fibers as compared with the WT retinas (Figures 2G,H).

### A2 Deletion Increases BDNF and GAP-43 Expression After ONC

**FIGURE 3:**
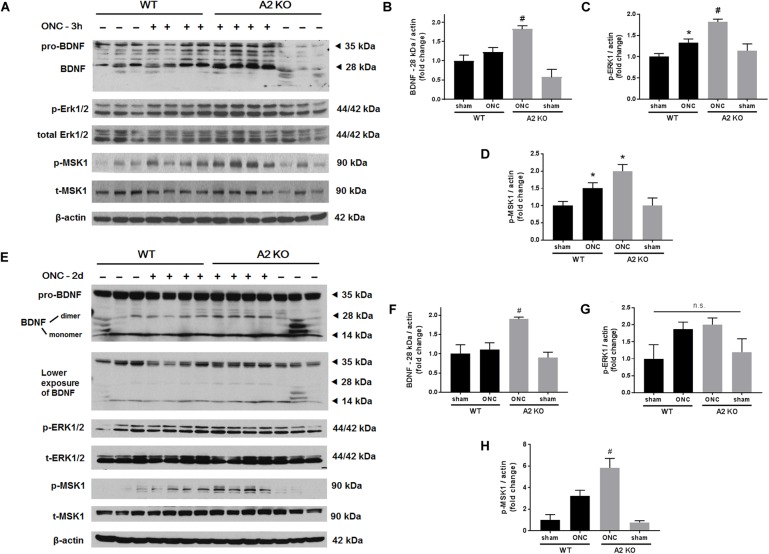
A2 deletion increases mature BDNF and its downstream signaling targets after ONC. Western blotting on retinal lysates and quantification at 3 h **(A–D)** and 2 days **(E–H)** after injury showed increased expression of mature BDNF (28 kD) and activation of its downstream signaling pathway (ERK/MSK) in the A2^-/-^ ONC retinas as compared to WT. *N* = 3–4, ^∗^*p* < 0.05 vs. WT sham, ^#^*p* < 0.05 vs. the other three groups, n.s. means no statistically significant difference.

**FIGURE 4:**
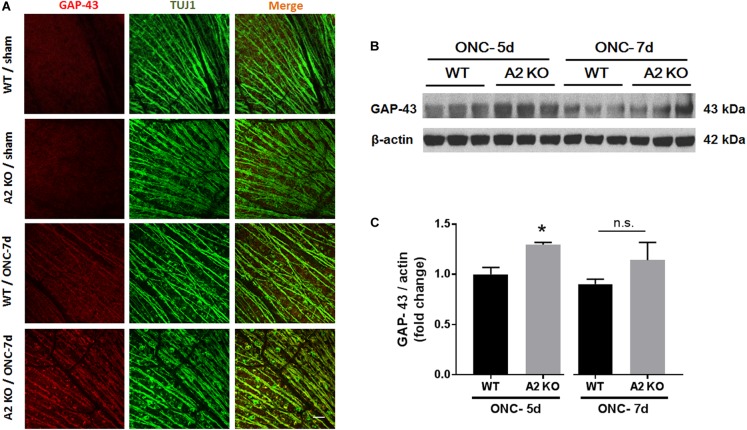
A2 deletion increases GAP-43 expression in nerve fibers after ONC. Flat mount immunolabeling **(A)** showed higher GAP-43 expression in nerve fibers of WT ONC vs. sham retinas at 7 days and A2 deletion further increased GAP-43 immunoreactivity. *N* = 4–5, scale bar = 50 μm. Western blotting on retina lysates **(B)** and quantification **(C)** showed increased GAP-43 expression in A2^-/-^ retinas as compared to WT that was significant at 5 days but did not reach statistical significance at 7 days after ONC. *N* = 3, ^∗^*p* < 0.05 vs. WT ONC, n.s. means no statistically significant difference.

Brain derived neurotrophic factor (BDNF) is a neurotrophin that has been shown to enhance the survival of RGC and their axons (Mysona et al., 2017). In order to examine BNDF involvement in the neuroprotective effect observed with A2 deletion, we measured BDNF levels using western blotting. A2^-/-^ retinas showed increased expression of BDNF (homodimer: 28 kDa) at 3 h after ONC as compared to WT retinas. This was associated with activation of BDNF downstream signaling in the A2^-/-^ retinas as shown by increased phosphorylation of extracellular signal–regulated kinase 1 (ERK1) and mitogen- and stress-activated protein kinase-1 (MSK1) (Figures 3A–D). Levels of BDNF and p-MSK1 but not p-ERK1 remained significantly elevated in the A2^-/-^ retinas at 2 days after ONC (Figures 3E–H). We also examined the expression of the axonal regeneration marker, growth associated protein (GAP)-43, using immunolabeling and western blotting. In line with increased BDNF expression, A2^-/-^ retina flat mounts showed increased GAP-43 labeling in nerve fibers as compared to WT at 7 days after ONC (Figure 4A). Western blotting on retina lysates confirmed an increase in GAP-43 in A2^-/-^ vs. WT retinas at 5 days after ONC with a similar trend at 7 days but the latter increase did not reach statistical significance. (Figures 4B,C).

### A2 Deletion Increases Axon Sprouting After ONC

**FIGURE 5:**
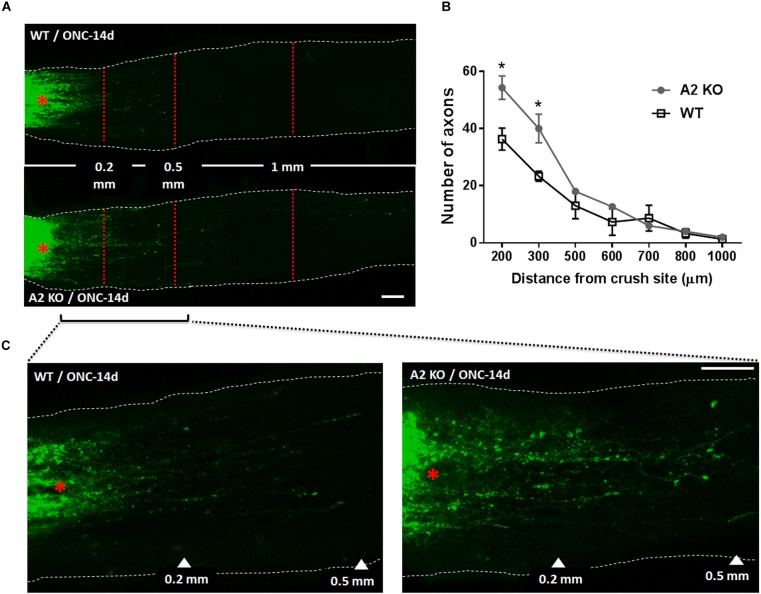
A2 deletion enhances axonal sprouting after ONC. Anterograde labeling of RGC axons using CTB **(A)** showed a significant increase in the number of axons that traveled beyond the crush site toward the brain at 200 and 300 μm distance from crush site denoted by asterisk **(B)**, *N* = 3 per group, ^∗^*p* < 0.05. **(C)** Shows higher magnification images of WT and A2^-/-^ optic nerves from crush site to 500 μm distance toward the brain. Scale bar = 100 μm.

At 14 days following ONC, RGC axons were labeled by anterograde transport of Cholera Toxin B (CTB) to examine the axonal sprouting and regrowth beyond the crush site and toward the brain. A2^-/-^ mice showed a statistically significant regenerative response manifested by increases in number of CTB-positive axons at 200 and 300 μm distance from the ONC site as compared with the WT controls (Figure 5).

### A2 Deletion Reduces Glial Activation and Retinal Inflammation After ONC

**FIGURE 6:**
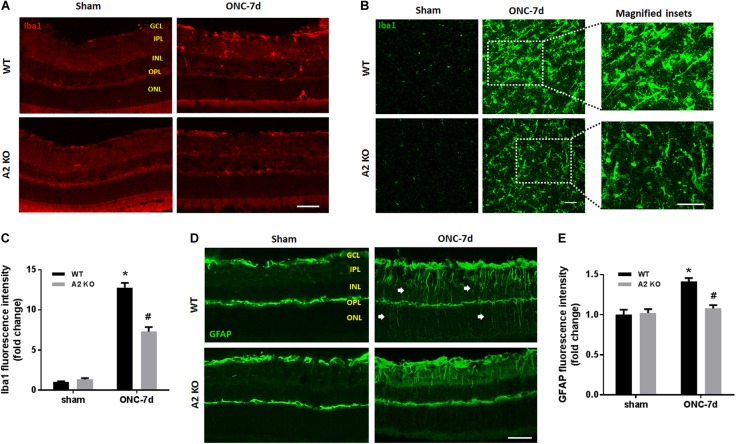
A2 deletion reduces glial activation after ONC. Immunolabeling for Iba1 in retina cross sections **(A)** and flat-mounts **(B)** together with quantification **(C)** showed prominent activation of microglia/macrophage (numerous Iba1^+^ cells with ameboid morphology) in WT retinas at 7 days after ONC. This was ameliorated in the A2^-/-^ retinas and the Iba1^+^ cells had a ramified morphology. *N* = 4. Labeling for GFAP on cross sections **(D)** and quantification **(E)** showed increased glial activation at 7 days after ONC which was reduced with A2 deletion. *N* = 5. ^∗^*p* < 0.01 vs. WT sham, ^#^*p* < 0.01 vs. WT ONC. Arrows denote upregulation of GFAP in the radial processes of activated Müller cells. Scale bar = 50 μm.

Müller cells and microglia/macrophages are activated following ONC (Mac Nair et al., 2016). To assess glial activation following ONC we labeled WT and A2^-/-^ retinas for Iba1 (microglia/macrophage marker) and GFAP (marker for Müller cell activation). WT retinas showed significant increases in immunoreactivity for both Iba1 and GFAP, suggesting an increase in activation of retinal microglia/macrophages and macroglial cells (Figure 6). In the Iba1 labeled retinal flat mounts from the ONC retinas the microglia had an ameboid morphology with rounded cell bodies and enlarged processes suggesting an activated phenotype (Figure 6B) and GFAP immunoreactivity extended from the GCL through the ONL in some areas, indicating activation of the Müller cells (Figure 6D). A2 deletion abated this increased activation as demonstrated by the smaller cell bodies and more ramified processes of the Iba1 labeled micrgoglia and decreases in fluorescence intensity for both Iba1 (Figure 6C) and GFAP (Figure 6E).

**FIGURE 7:**
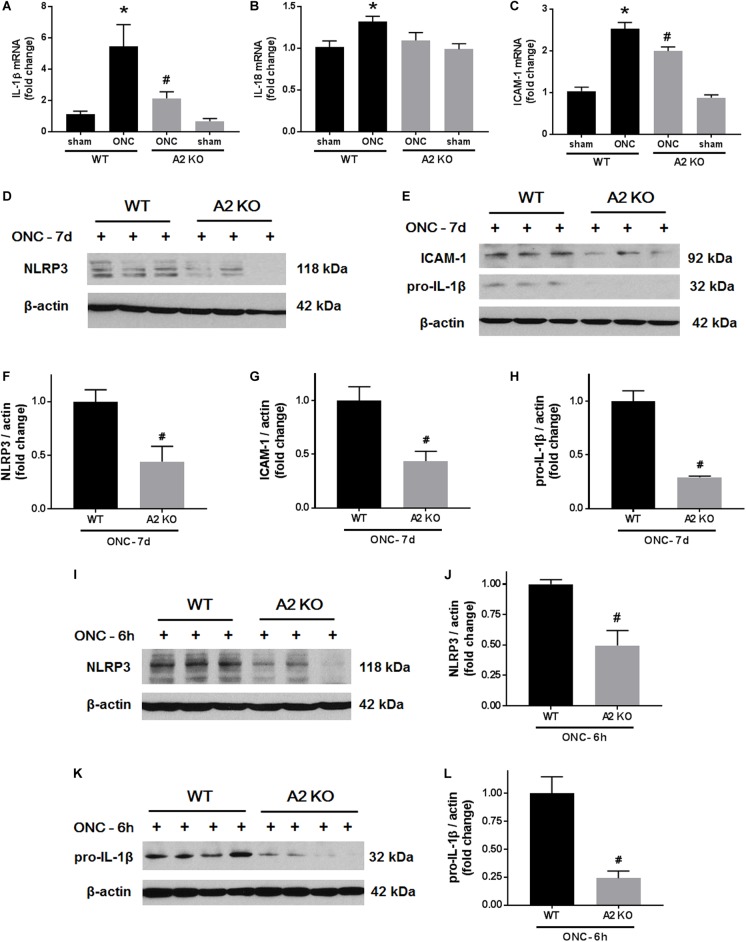
A2 deletion ameliorates ONC-induced retinal inflammation. Quantitative RT-PCR **(A–C)** showed upregulation of pro-inflammatory mediators (IL-1β, IL-18, and ICAM-1) at 7 days after ONC in the WT retinas. A2 deletion significantly blunted these increases. *N* = 4–6, ^∗^*p* < 0.05 vs. WT sham, ^#^*p* < 0.05 vs. WT ONC. Western blotting at 7 days after ONC **(D,E)** and quantification **(F–H)** showed a significant reduction in NLRP3, ICAM-1, and pro-IL-1β protein levels in A2^-/-^ retinas as compared to WT. Western blotting on retina lysates **(I,K)** and quantification **(J,L)** showed downregulation of NLRP3 and pro-IL-1β in A2^-/-^ retinas as compared to WT at 6 h after ONC as well. *N* = 3–4, ^#^*p* < 0.05 vs. WT ONC.

We next examined the effects of A2 deletion on markers of retinal inflammation after ONC injury. RT-PCR analysis showed significant increases in mRNA levels for interleukin (IL-) 1β, IL-18, and the intracellular adhesion molecule (ICAM-1) at 7 days after ONC injury. Each of these changes was significantly inhibited in the A2^-/-^ retinas (Figures 7A–C). We used western blotting to confirm these suggested protective effects of the A2 deletion at the level of protein expression at 7 days after the injury. This analysis verified the effect of the A2 deletion in decreasing the nucleotide-binding domain (NOD)-like receptor protein 3 (NLRP3), pro-IL-1β and ICAM-1 protein levels (Figures 7D–H). Western blot analysis of samples collected at 6 h after ONC also showed a significant protective effect of A2 deletion in reducing levels of NLRP3 and pro-IL-1β (Figures 7I–L).

### A2 Deletion Suppresses Apoptosis and Preserves Retinal Thickness After ONC

**FIGURE 8:**
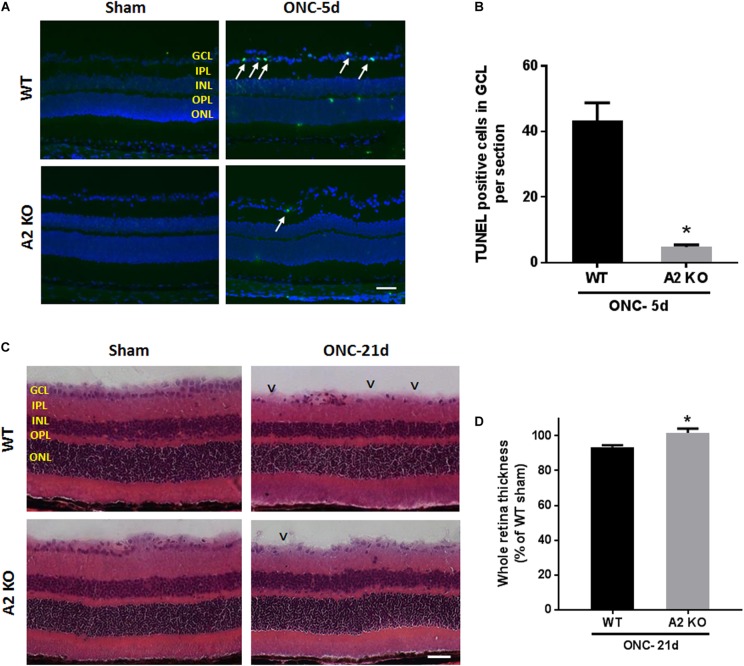
A2 deletion ameliorates ONC-induced apoptosis and limits retinal thinning. TUNEL labeling **(A)** of retina cross sections and quantification **(B)** showed increased TUNEL positive cells in the GCL (arrows) at 5 days after ONC. A2^-/-^ retinas showed a significant reduction in TUNEL positive cells. *N* = 4–6 per group, ^∗^*p* < 0.01, scale bar = 50 μm. Hematoxylin and eosin (H&E) staining of retina cross sections **(C)** and quantification **(D)** at 21 days showed that A2 deletion preserved the total retina thickness to near sham levels. *N* = 6 per group, ^∗^*p* < 0.01, scale bar = 50 μm. Arrowheads denote area of RGC loss.

ONC induces apoptotic cell death of RGC that peaks within the first week as measured by TUNEL staining (Li et al., 1999). To examine the potential role of A2 expression in ONC-induced apoptosis we implemented TUNEL staining on WT and A2^-/-^ retina sections at 5 days after ONC. A2 deletion markedly suppressed ONC-induced apoptosis as shown by a marked decrease in numbers of TUNEL positive cells in the GCL as compared with the WT retinas (Figures 8A,B). Furthermore, ONC injury induces thinning in the inner retina layers starting at 7 days and reaching a steady level at 14–28 days (Liu et al., 2014; Yukita et al., 2015). Our analysis of retina thickness showed improved preservation of retinal layer morphology in the A2^-/-^ mice as compared with the WT mice at 21 days after ONC. A2^-/-^ retinas showed reduced thinning of the whole retina as compared to WT retinas subjected to ONC (Figures 8C,D).

## Discussion

In this study, we have investigated the role of A2 in TON by studies using a mouse model of ONC. Our results show that A2 is upregulated within hours of ONC-injury. Deletion of A2 results in a delay in RGC loss and axonal degeneration together with increased expression of the regeneration marker GAP-43 and increased axonal sprouting. Furthermore, A2 deletion was associated a reduction in inflammatory mediators, a decrease in numbers of TUNEL positive cells and diminished activation of Müller cells and microglia.

ONC-injury induces progressive RGC death, which starts as early as 7 days and increases with time (Liu et al., 2014). We examined RGC loss using NeuN (general marker for neurons), Brn3a and RBPMS (specific markers for RGC). Some reports suggest that expression of Brn3a can be downregulated in RGC after injury (Nuschke et al., 2015). Therefore, we used the other markers to confirm our results. The relative percentage of lost RBPMS-positive cells at 14 days was slightly less than that of the Brn3a-positive cells, which is consistent with possible downregulation of Brn3a after RGC injury. Moreover, since NeuN labels displaced amacrine cells as well as RCGs, the relative percentage of lost NeuN-positive cells was less as compared to Brn3a at all the time points examined. While the neurodegeneration progressed with time in both WT and A2^-/-^ retinas, A2 deletion resulted in significant neuronal preservation as compared to WT when evaluated using either marker.

In addition to the neuronal loss, WT retinas showed axonal degeneration after ONC as measured by immunolabeling of GCL axons with TUJ1 at 7 and 14 days. A2^-/-^ retinas showed axonal preservation. This protective effect was associated with increases in axonal sprouting as shown by anterograde transport of CBT at 14 days after ONC injury and increased expression of the regeneration marker, GAP-43 at 5 and 7 days. GAP-43 is a developmental growth protein that also plays an important role in axonal regeneration and plasticity in the adult nervous system (Holahan, 2017). It is induced after ONC injury (Doster et al., 1991), and is further upregulated by BDNF administration (Fournier et al., 1997; Klocker et al., 2001). GAP-43 is an important mediator of the neuroprotective effects of BDNF (Gupta et al., 2009). In line with this, our molecular analysis showed an early increase in BDNF protein together with activation of its downstream signaling pathway (ERK/MSK) in the A2^-/-^ retinas after ONC as compared to WT. BDNF (monomer: 14 kDa, homodimer: 28 kDa) is a neurotrophin that results from cleavage of its precursor protein, pro-BDNF (35 kDa). BDNF has been shown to be expressed in RGC and glia in adult rodent retinas. Several reports have shown that BDNF is an important mediator of RGC survival and axonal preservation (Mysona et al., 2017). To our knowledge, our results are the first to show a link between A2 and BDNF. Further studies are needed to address the mechanism by which A2 deletion increases BDNF levels after ONC and the cellular sources involved in the process.

Our previous studies in mouse models of OIR and retinal IR injury have shown decreased glial activation with A2 deletion (Narayanan et al., 2011; Shosha et al., 2016). Glial activation is a hallmark of ONC injury and has been implicated in its pathogenesis. ONC stimulates both macroglia and microglia with peak activation at 7 days (Mac Nair et al., 2016). Our results show that A2 deletion ameliorates glial activation as indicated by reductions in GFAP and Iba1 labeling. Furthermore, A2 deletion dampened the ONC-induced retinal inflammation as measured by decreases in the NLRP3 inflammasome, ICAM-1 and inflammatory cytokines IL-1β/IL-18. The NLRP3 inflammasome has been shown to be upregulated in retinal microglia after ONC. This upregulation in NLRP3 was suggested to mediate the RGC and axonal loss after ONC (Puyang et al., 2016). It is suggested that the inflammatory response and glial activation elicit secondary neurodegeneration after ONC, whereby RGC with intact axons undergo apoptosis due to the ongoing inflammation (Li et al., 2014; Mac Nair et al., 2016). Therefore, A2 deletion-mediated dampening of retinal inflammation and glial activation is likely to play a role in RGC preservation and axonal sprouting.

Apoptosis, demonstrated by DNA fragmentation, has been described as the mechanism of RGC death after ONC-injury. TUNEL positive cells peaked at 7 days after ONC (Li et al., 1999). Our data showed that A2^-/-^ retinas exhibited less TUNEL positive cells at 5 days as compared to WT. The decrease in apoptotic cells in A2^-/-^ retinas can be attributed to the upregulation in BDNF levels and decreased retinal inflammation leading to increased RGC resistance to ONC damage. Similar to the RGC preservation, A2^-/-^ retinas showed a reduction in retinal thinning as compared with the WT retinas at 21 days after ONC.

## Conclusion

In conclusion, our study shows an important role of A2 expression in mediating ONC pathology. A2 deletion ameliorates RGC apoptotic cell death and increases regeneration markers, BDNF and GAP-43. Targeting A2 could provide a new therapy for traumatic injury to the optic nerve.

## Author Contributions

RBC managed and funded the project. RBC, RWC, and SPN conceptualized and designed the study, crticially revised, and edited the manuscript. ZX, TL, ES, MR, FL, and CP performed the experiments. ZX and AF analyzed and interpreted the resutts, prepared the figures, and drafted the manuscript.

## Conflict of Interest Statement

The authors declare that the research was conducted in the absence of any commercial or financial relationships that could be construed as a potential conflict of interest.

## Abbreviations

A2: arginase 2
A2^-/-^ or A2 KO: A2 knockout
GCL: ganglion cell layer
H&E: hematoxylin and eosin
INL: inner nuclear layer
IPL: inner plexiform layer
OCT: optimal cutting temperature
ONC: optic nerve crush
ONL: outer nuclear layer
OPL: outer plexiform layer
PFA: paraformaldehyde
RGC: retinal ganglion cells
TON: traumatic optic neuropathy
WT: wild type
